# Emerging advances in biosecurity to underpin human, animal, plant, and ecosystem health

**DOI:** 10.1016/j.isci.2023.107462

**Published:** 2023-07-31

**Authors:** Philip E. Hulme, Jacqueline R. Beggs, Rachelle N. Binny, Jonathan P. Bray, Naomi Cogger, Manpreet K. Dhami, Susanna C. Finlay-Smits, Nigel P. French, Andrea Grant, Chad L. Hewitt, Eirian E. Jones, Phil J. Lester, Peter J. Lockhart

**Affiliations:** 1 The Centre for One Biosecurity Research, Analysis and Synthesis, Lincoln University, PO Box 85084, Lincoln, Christchurch 7648, New Zealand; 2 Department of Pest Management and Conservation, Lincoln University, PO Box 85084, Lincoln, Christchurch 7648, New Zealand; 3 Centre for Biodiversity and Biosecurity, School of Biological Sciences, University of Auckland, Private Bag 92019, Auckland 1142, New Zealand; 4 Manaaki Whenua - Landcare Research, PO Box 69040, Lincoln, New Zealand; 5 Tāwharau Ora, School of Veterinary Science, Massey University, Palmerston North 4472, New Zealand; 6 Scion, 10 Kyle Street, Riccarton, Christchurch 8011, New Zealand; 7 School of Biological Sciences, Victoria University of Wellington, PO Box 600, Wellington, New Zealand; 8 School of Natural Sciences, Massey University, Palmerston North 4472, New Zealand

**Keywords:** Natural sciences, Ecology, Biological sciences

## Abstract

One Biosecurity is an interdisciplinary approach to policy and research that builds on the interconnections between human, animal, plant, and ecosystem health to effectively prevent and mitigate the impacts of invasive alien species. To support this approach requires that key cross-sectoral research innovations be identified and prioritized. Following an interdisciplinary horizon scan for emerging research that underpins One Biosecurity, four major interlinked advances were identified: implementation of new surveillance technologies adopting state-of-the-art sensors connected to the Internet of Things, deployable handheld molecular and genomic tracing tools, the incorporation of wellbeing and diverse human values into biosecurity decision-making, and sophisticated socio-environmental models and data capture. The relevance and applicability of these innovations to address threats from pathogens, pests, and weeds in both terrestrial and aquatic ecosystems emphasize the opportunity to build critical mass around interdisciplinary teams at a global scale that can rapidly advance science solutions targeting biosecurity threats.

## Graphical abstract

**Figure:**
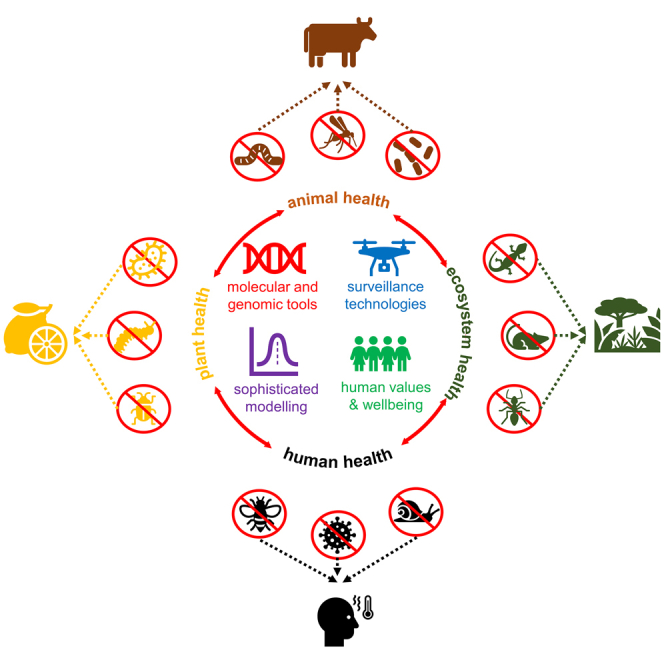

## Introduction

Invasive alien pathogens, pests, and weeds pose a significant challenge to the environment worldwide and their management results in huge global economic costs.^1^^,^^2^ Biosecurity covers all activities aimed at managing the introduction of alien species to a particular region and mitigating their impacts should they become established. It includes the implementation of international sanitary and phytosanitary standards, border inspection, post-border surveillance, incursion response and long-term management of invasive alien pathogens, pests, and weeds.^3^^,^^4^ Since the goal of biosecurity is the exclusion, eradication or effective management of risks posed by introduced pests and diseases to the economy, environment and human health, it is fundamentally an interdisciplinary activity that underpins human, animal, plant, and ecosystem health (see Box 1 for definitions).^5^^,^^6^

Box 1 **Definitions of key terms addressing human, animal, plant and ecosystem health**

Human Health: Consistent with the World Health Organization, this covers the prevention and protection from human communicable diseases including anthroponoses (when the source is an infectious human), zoonoses (the source is an infectious animal), and sapronoses (the source is an abiotic substrate, non-living environment) and includes pathogens, prions and parasites. It also includes human wellbeing including protection from non-infectious diseases, food-borne hazards and immunological mediated hypersensitivity (asthma and allergy).

Animal Health: Consistent with the World Organization for Animal Health (WOAH) it includes the prevention and management of epizootic diseases that affect terrestrial and aquatic animals, including wildlife as well as animal welfare. This includes an understanding of invasive species as vectors of animal diseases but also their detrimental role in animal welfare through harm because of bites, stings, poisoning, dermatitis, photosensitization etc.

Plant Health: Consistent with the International Plant Protection Convention, plant health is an overarching term for emerging risks including pests, diseases and weeds, integrated pest management and innovation in plant protection. The definition extends beyond the protection of cultivated plants to the protection of natural flora and plant products. It also takes into consideration both direct and indirect damage by pests, so it includes weeds.

Ecosystem Health: Ecosystem health is a holistic measure describing the general condition of an ecosystem in relation to the impacts of environmental change drivers such as pollution, overharvesting, land-use change, climate change and the effects of alien pathogens, pests, and weeds. Although there is no standard benchmark as to what qualifies as a healthy ecosystem, the term is frequently applied to portray the state of ecosystems in relation to their conservation status. In the context of the present article, the emphasis on ecosystem health is primarily on the factors influencing the distribution, population dynamics and impacts of invasive alien species.

One Health: One Health is a collaborative, multisectoral approach that aspires to sustainably balance and optimize the health of people, animals, and ecosystems. By recognizing that the health of humans, animals, plants, and the wider environment are closely linked and interdependent, One Health aims to achieve optimal health and well-being outcomes.

**Figure 1:**
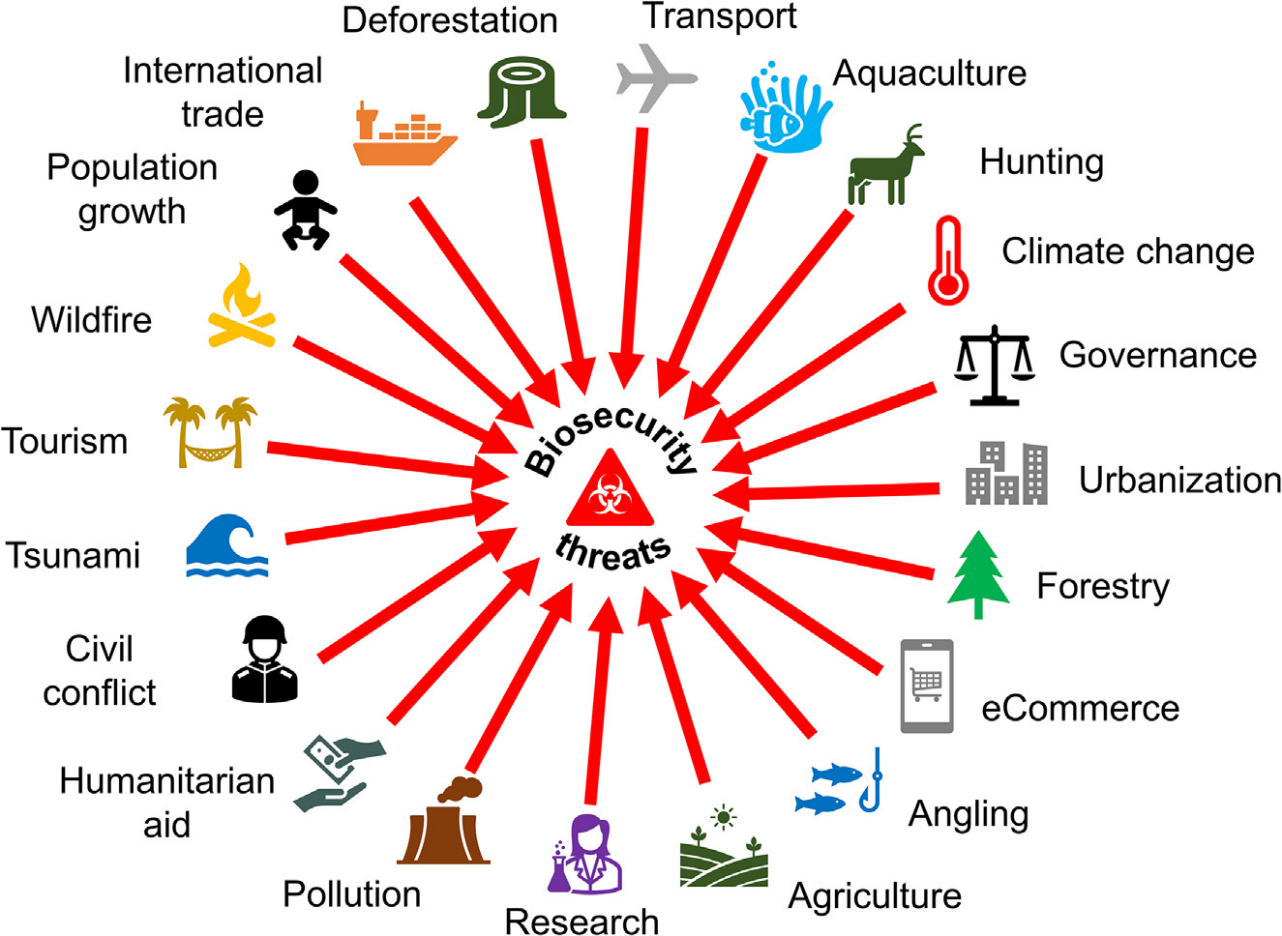
Schematic illustrating the diversity of environmental, economic, and social drivers of biological invasions^13^ Each of the drivers depicted in the figure can lead to the introduction of invasive alien pathogens, pests, or weeds that impact human, animal, plant, and ecosystem health.

Nevertheless, despite fundamental similarities in the invasion process of pathogens, pests, and weeds, irrespective of whether they impact human, animal, or plant health, government policymakers and regulators, researchers, and industry generally take a siloed approach to biosecurity delimited by sectoral and taxonomic identities. Although there have been calls to better integrate invasive alien species threats under a One Health umbrella,^7^^,^^8^ there is increasing recognition that One Health remains largely focused on interactions between human and animal health, especially in relation to zoonoses, while plant and ecosystem health considerations remain poorly integrated in terrestrial systems and are virtually absent in aquatic contexts.^9^^,^^10^^,^^11^ Similarly, the importance of social aspects of biosecurity and disease management appear to be routinely ignored in the narrative of One Health.^12^ Yet many drivers of biological invasions that affect human, animal, plant, as well as ecosystem health are societal issues such as poor governance, human population growth, urbanization, leisure and tourism, international trade, or civil conflict and the relative contributions of these socioeconomic drivers remain poorly quantified (Figure 1).^13^

In response, the concept of One Biosecurity has been developed to foster an interdisciplinary approach to biosecurity policy and research, building on interconnections between human, animal, plant, and ecosystem health to prevent and mitigate the impacts of invasive alien species.^14^ The development of the One Biosecurity concept to date has primarily focused on describing: (1) the cross-sectoral impacts of many invasive alien species (e.g., fire ants, rats) that have negative impacts across the human, animal, plant, and ecosystem health sectors; (2) the multisectoral threat posed by global change drivers (e.g., climate change, agricultural intensification, urbanization) that increase the risk of biological invasions; and (3) the international policy context of dealing with pandemic threats to human, animal, plant, and ecosystem health posed by invasive alien species.^11^^,^^14^ However, a key component of the biosecurity system is the science community, and within this community there is a need for a unified research approach that underpins the detection, prevention, and management of biological invasions. By identifying recent innovations that are cross-sectoral, opportunities exist to build critical mass around interdisciplinary teams to rapidly advance science solutions targeting biosecurity threats. But what are the major emerging issues? To address this question, a structured, Delphi method was adopted to scan the horizon for innovative research underpinning the future of human, animal, plant, and ecosystem health (Box 2). Drawing on the diverse biosecurity expertise of participants enabled the identification and critical discussion of four emerging issues that are relevant to biosecurity irrespective of the sector, invasive organism or biome concerned.

Box 2 **Identifying emerging issues underpinning One Biosecurity**

**Figure:**
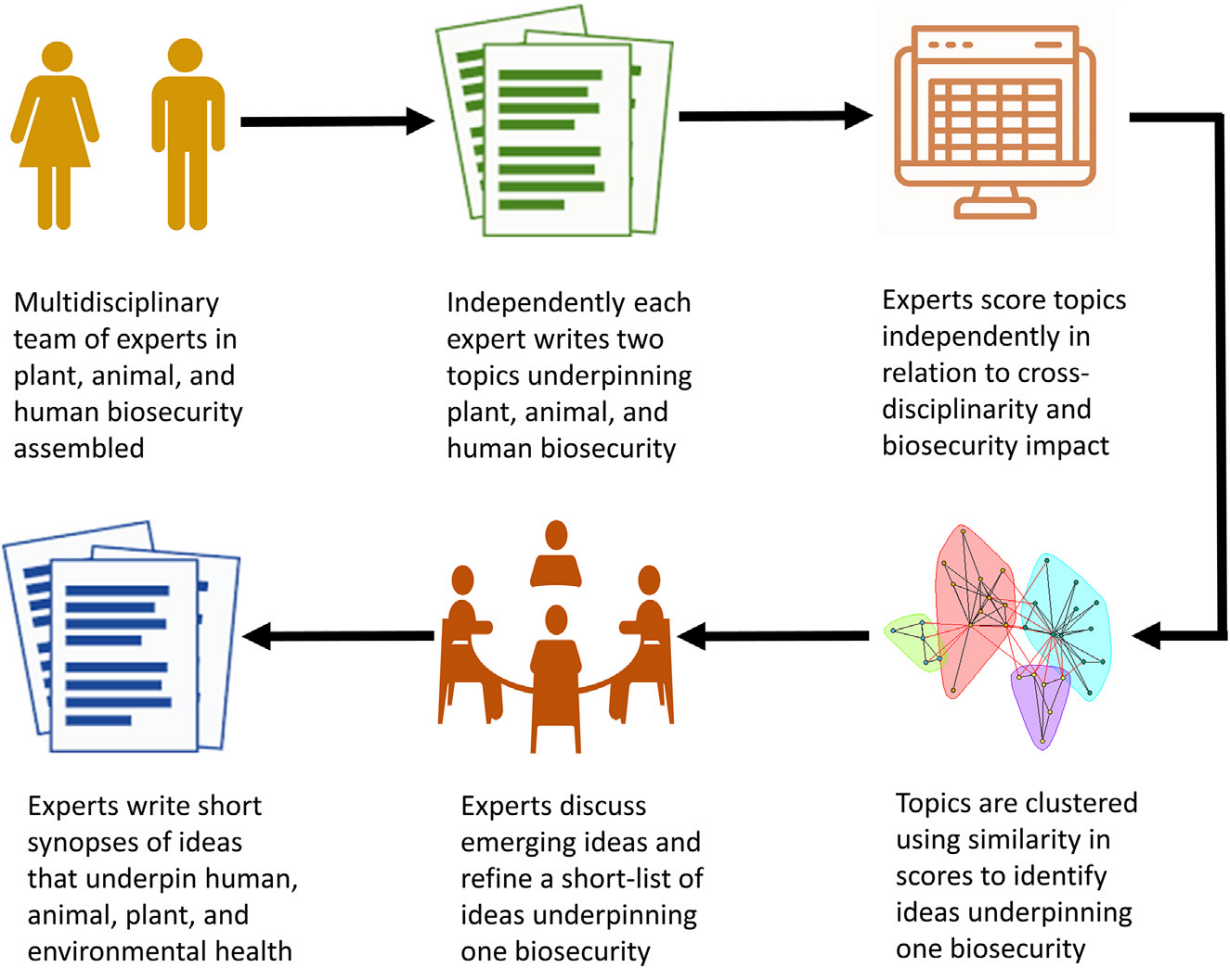

Emerging biosecurity topics were identified and evaluated using a modified iterative Delphi method of expert consultation such as voting and anonymity, similar to procedures used in horizon scans of biological invasions.^15^^,^^16^ An interdisciplinary team of human, animal, and plant biosecurity specialists was assembled bringing together a varied skill set encompassing epidemiology, genomics, invasion biology, modeling, pathology, pest management, social sciences and veterinary science across the terrestrial and aquatic biomes (6 male and 7 female). Each participant was asked to submit at least two short (200–300 word) synopses describing emerging cross-sectoral biosecurity issues (See Supplementary Material online for full list). These submitted topics were circulated to all members, each of whom independently scored them (1–5) against three criteria: the scope of influence, potential impact, and degree of novelty. Topics were then clustered using their similarity in scores across all participants. Emerging issues were identified where topics formed clearly distinguishable clusters. These emerging issues were discussed and agreed at the workshop and further elaborated to maximize their scientific impact.

## Emerging advances in biosecurity

### Innovative autonomous surveillance technologies for biosecurity

Conventional surveillance of biosecurity incursions for invasive alien pathogens, pests, and weeds using traps or direct observation is labor intensive, time-consuming, costly, and rarely completely effective. Although across the human, animal, plant, and ecosystem health sectors there is growing interest in using the keen sense of smell possessed by dogs to detect chemical signatures associated with human pathogens^17^ as well as invasive alien plants, pest insects and mammals,^18^^,^^19^^,^^20^ future advances in biosecurity will undoubtedly rely on more reliable and deployable novel sensor technologies. Ultra-fast gas chromatography (e-nose) can be used to analyze volatiles released by pests (e.g., kairomones, pheromones) or chemicals released when plant or animal tissues are degraded by pathogens.^21^ However, since biosecurity threats are spatially dynamic processes, sensors need to be deployed over large areas and be sufficiently mobile to track the movement of invasive alien species. Many of the challenges facing the application of surveillance systems in biosecurity monitoring for human, animal, plant, and ecosystem health, including spatial resolution and coverage, duration, and temporal scale, can be resolved by using autonomous systems.

Autonomous surveillance technologies, such as sensor networks and robotic systems, linked to intelligent algorithms, have the potential to prevent, detect and manage biosecurity threats. For example, sensing and robotic systems have been used to track flying foxes as disease vectors; detect fruit flies, identify weeds, and differentiate diseased from healthy plants.^22^ During the SARS-CoV-2 pandemic, high-speed thermal-metric monitors were used to scan and instantly identify individuals with a fever in a moving flow of people, although it was later found to be ineffective in identifying infectious individuals and limiting the spread of disease.^23^ Nevertheless, there is an opportunity to build on some of these developments that initially targeted human diseases to improve detection of animal and plant diseases (e.g., high temperature related to infection), and to develop other technologies/systems that work across common biosecurity threats for human, animal, plant, and ecosystem health.^24^^,^^25^ Visual sensor networks in combination with artificial intelligence algorithms can be used to identify insects in pheromone traps, weeds and diseased plants in cropping and natural ecosystems, as well as aquatic biofouling organisms on the hulls of ships.^26^^,^^27^ There is also potential to combine multiple layers of autonomous surveillance technologies into integrated systems for targeted detection, localization, and management of biosecurity incursion threats. Thus, an aerial drone could not only sense and identify weeds, pests, or diseased plants, but also deliver targeted management through the application of herbicides, pesticides, or fungicides.

A case in point is the Internet of Things (IoT) that refers to the network of sensors that connect and exchange data with other devices and systems over the Internet or other communications networks. While the application of IoT is still in its infancy, it has considerable scope to improve the tracing and tracking of biosecurity risks. Passive radio frequency identification devices (RFID) store information on a microchip that, when combined with a suitable mobile receiver, permit out-of-line-of-sight tracing and have been used to track the movements of shipping containers, livestock and pets, horticultural plants and even hospital patients.^28^^,^^29^ Animals and plants (as well as their associated pathogens) found to be posing a biosecurity threat can be traced back to their origin by scanning RFID tags at specific locations e.g., farms, sales yards, abattoirs, and horticultural nurseries (for plants and livestock), ports, devanning locations, warehouses (for shipping containers).^30^^,^^31^ Similarly, if tagged livestock (such as goats or pigs) and even pets escape confinement to become feral then following capture, the tags can be read, and the source identified.

More recently, Bluetooth Low Energy (BLE) sensors have been developed with a battery life of up to 5 years, and these are able to send an electromagnetic signal at distances of over 200m. Because Bluetooth devices can communicate with each other, they can capture details of similar devices nearby and thus it is possible to track organisms, people or containers, allowing checks as to whether there has been contact with a biosecurity incursion or risk, enabling more comprehensive tracing.^32^^,^^33^ Bluetooth devices on mobile phones have also been used to trace the proximity of people to individuals with COVID-19 symptoms. Uptake of this technology was initially poor due to privacy concerns but it also performed poorly when the pandemic was in full swing due to the high frequency of alerts described as a “pingdemic”.^34^ However, earlier rollout of such automated tracing technology and close alignment with manual contact tracing efforts, might have been effective for reducing transmission in the initial stages of the pandemic.^35^ The application of the IoT technologies in human, animal, plant, and ecosystem health highlights the opportunities for better collaborative approaches across sectors to develop more reliable sensors. Given the huge volumes of data generated, and the need to integrate multiple data sources, further development of the IoT within the field of biosecurity would also be necessary.

### Molecular and genomic tools for biosecurity surveillance

The rapidly evolving field of nucleic acid based environmental metabarcoding (eNA) has led to an unprecedented ability to identify potential biosecurity threats to human, animal, plant, and ecosystem health. There is a huge opportunity to leverage the rapid development of eNA diagnostic tools used during the SARS-CoV-2 pandemic to improve the detection of biosecurity threats in the animal, plant, and ecosystem health sectors. Rapid, sensitive, and cost-effective tools include loop-mediated isothermal amplification (LAMP) and recombinase polymerase amplification (RPA) which have recently been implemented as portable devices that can be deployed in the field for “point of care” detection.^36^^,^^37^ Recent biosecurity applications of LAMP and RPA include detection of pathogens and insect pests of plants,^38^^,^^39^ parasites and pathogens of livestock,^40^^,^^41^ zoonotic pathogens,^42^ SARS-CoV-2 in human wastewater^43^ and invasive alien species in freshwater and marine ecosystems.^44^^,^^45^

Despite differences in the gene targets that are used for taxonomic resolution, the application of eNA in human, animal, plant, and ecosystem health faces similar challenges including sampling and purifying nucleic acids from environmental samples, incomplete reference databases, and complex bioinformatics requirements that might better be resolved by an integrated approach across the different sectors.^46^^,^^47^ A major benefit of eRNA over eDNA surveillance is the ability to distinguishing the living portion of a community, which is essential to confirm that biosecurity treatments are effective, but also to target surveillance efforts where populations of invasive pests and pathogens are growing.^46^ By the same token, recent developments in the diagnostics of airborne and soil pathogens of agriculture could be extended to inform approaches for studies of human pathogens.^48^ Thus, while eNA tools are increasingly applied in environmental monitoring, considerable potential exists to deliver diagnostics across human, animal, plant, and ecosystem health. There remains considerable potential of linking these technologies to develop passive sensors that can be combined with autonomous surveillance tools.

Furthermore, eNA has been recognized as a cost-effective tool to monitor evolution and has been used to track the changes of nucleotide diversity, derive variant-specific reproduction numbers, and geographically locate the emergence of novel mutation constellations in human pathogens.^49^ Pathogens can adapt dramatically over short periods of time, so eNA monitoring could identify the evolution of new epidemic strains through events such as recombination in near real-time.^50^ This selection and pathogen evolution is most clearly demonstrated with environmental selection for antibiotic resistance in bacterial pathogens. The presence of antibiotics in wastewater can be a substantial on-site selection pressure representing an increased risk that bacteria may acquire resistance genes directly through eNA uptake.^51^

The ability to track evolutionary change using high-throughput third generation nucleic acid sequencing, provides the capacity to sequence, analyze, and interpret data in near real-time and at relatively low cost, also offers an opportunity to determine the geographical origin of biosecurity incursions, and their subsequent spread post-border. Mitochondrial and genomic metabarcoding have been used to support phylogeographic analyses that describe the origin and global spread of a wide range of invasive alien pests and pathogens.^52^^,^^53^^,^^54^^,^^55^ Full genome sequencing provides further opportunities for phylodynamic modeling, that explores the joint dynamics of epidemiological and evolutionary processes to determine the origin and spread of human, animal, and plant pathogens.^56^^,^^57^^,^^58^ Phylodynamic modeling has made significant progress in recent years due to increasingly available genomic data and advances in statistical modeling but there remain many challenges to the widespread application of phylodynamic models for biosecurity. These challenges include accounting for evolutionary complexities such as changing mutation rates, selection, reassortment, and recombination, as well as epidemiological complexities such as stochastic population dynamics, and host population structure.^59^ Advances in bioinformatics and phylodynamic modeling, coupled with metagenomics, could help determine the most likely time, origin, and frequency of multiple biosecurity incursions, as well as subsequent spatial and temporal dynamics, and be an important element of a biosecurity system.

### Incorporating human values and wellbeing into biosecurity decision-making

Governments are increasingly looking to enhance public participation in biosecurity since greater engagement of wider ranging actors can benefit invasive alien species surveillance and detection, and participation in eradication, control programs, and prevention activities.^60^^,^^61^ Supporting greater involvement of people in biosecurity requires attention to the needs, motivations, and interests of people in different settings where biosecurity measures are implemented.^60^ A perceived lack of personal relevance of biosecurity may be a major stumbling block in engaging with communities (Box 3). All decision-making processes require a choice between alternative options based on their perceived merits and drawbacks that may reflect corporate, economic, cultural, ethical, or spiritual values. The basis for decision-making and priority setting in biosecurity is not always clearly articulated but can often be driven by expert-led science and economic rationalism. However, to engender effective public and stakeholder compliance with biosecurity measures and/or engagement with biosecurity responses, decision-making should also consider a broader set of psychological, social and cognitive attributes of the affected individuals and communities to guide policies, practices, and decisions.^64^

Box 3 **Who is responsible for biosecurity?**

**Figure:**
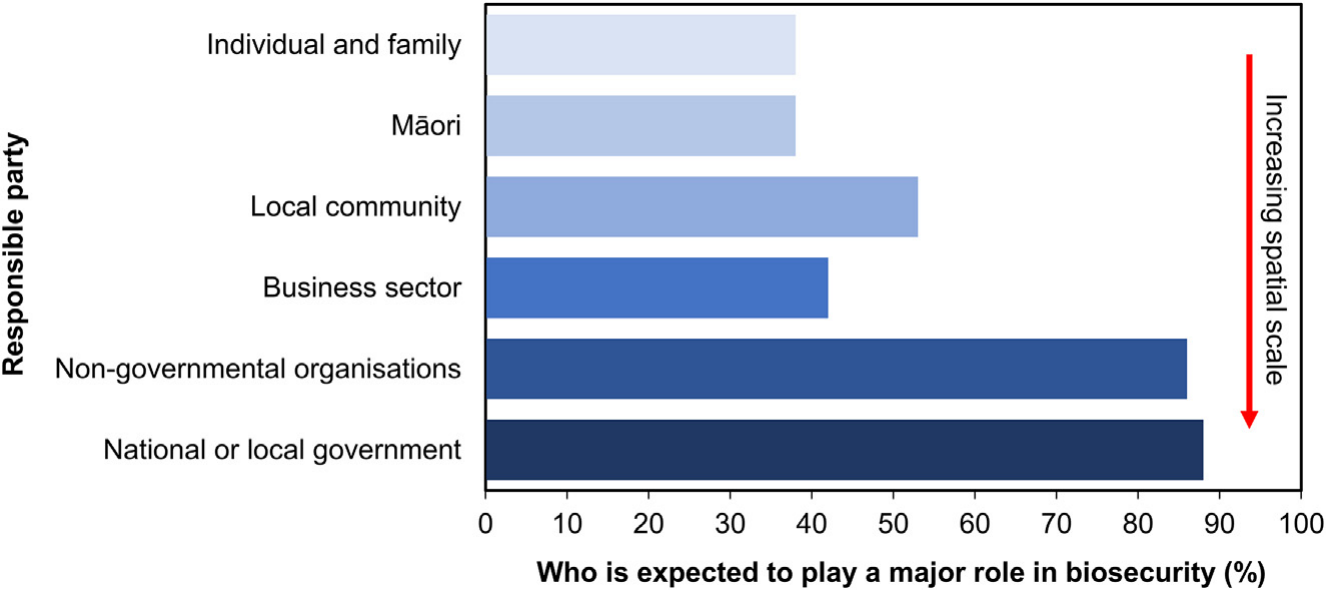

In 2018, the New Zealand government sponsored a survey to capture public attitudes toward biosecurity. The survey comprised a representative (in relation to age, gender, and socioeconomic status) sample of 1150 adults, including a specific sample of 150 Māori (indigenous people of New Zealand). The survey highlighted that most individuals do not see themselves or their family playing a substantial role in the biosecurity system and instead identify national and local government as the main players in the protection of the environment and economy from invasive alien pathogens, pests, and weeds.^62^ The survey also suggested that while much of the public in New Zealand (96%) see biosecurity as an important safeguard for the protection of the environment and economy, only 2% specifically think of the consequences to themselves and their way of life. This perspective was particularly strong in the respondents under 30 years old. These results echo with a more focused survey addressing responsibilities for managing invasive alien species in New Zealand coasts and beaches, which found respondents placing responsibility with national and local government, followed by business and finally individual members of the public.^63^ This perceived lack of personal relevance and reliance on government actions may be a major stumbling block in engaging communities with biosecurity, resulting in a shift in decision-making toward one set of values and likely prioritizes economic rather than environmental sectors.

Thus, the characteristics of any biosecurity policy or response should identify the perceived merit of the system at risk whether the system is human society, a semi-natural landscape, an ecosystem, a farm, or even a single species. Assessments of merit should take into account the inherent worth of the system, the services (financial or cultural) it provides to people, and/or the meaningfulness of relationships between people and the system at risk, such as cultural identities of indigenous people derived from their connection to the land.^65^ Environmental, economic, social, and cultural value systems are derived from various sources covering a wide suite of domains including both evidence-based understanding and perceptual belief. Engagement methodologies for ensuring minimum disruption to local cultural practices or their adaptation with new invasive alien species are required to ensure community preferences and practices are integrated into any management plans.^66^

The inclusion of indigenous communities and local users of environments who have previously been excluded from biosecurity decision-making processes must, however, be meaningful, involving an equitable sharing of power, influence, and resources.^67^ Broad public engagement can require levels of trust developed through dialogues to ensure concerns can be addressed. ^68^^,^^69^Trust is particularly important under conditions of uncertainty where biosecurity threats are less visible or distant, and when precautions taken to prevent transport of pests or pathogens impact other desirable or routine activities.^70^^,^^71^ To achieve a higher level of engagement in biosecurity requires that people are aware of the consequences of not protecting their environment, whether natural or anthropogenic, against an increasing number of threats to human, animal, plant, and ecosystem health. A greater awareness of the role played by people in these environments is needed to decrease exposure to biosecurity risks.

The direct effects of biosecurity incursions on human, animal, and plant health have often been quantified in economic terms^2^ but less well appreciated is the indirect effects such biosecurity responses have on general wellbeing of the human population. Outbreaks of human, animal, and plant pathogens often result in the implementation of formal restrictions on the movement of people, particularly into natural areas to prevent the spread of pests or pathogens.^72^^,^^73^^,^^74^^,^^75^ Although pesticide use has undoubtedly brought economic benefits in agricultural production, unintended exposure to humans can be extremely hazardous.^76^ Thus the deployment of insecticides, herbicides, or toxic bait over large areas, can result in the subsequent avoidance of these areas by the public until the operations have been completed.^77^ Yet exposure to nature has numerous benefits for human health and wellbeing, including improvements in mental health and stress reduction, cognitive function, physical health, social wellbeing, and self-control.^78^ The response to biosecurity threats often considers the direct health and safety risk to the public as well as public acceptability of the response but rarely the indirect effects on human wellbeing.

There is, thus, a need to understand the benefits and impacts of biosecurity actions on the wellbeing of people both directly and indirectly. Appreciating what is at risk from biosecurity incursions also requires attention to how people are impacted not only in terms of loss of livelihoods (e.g., farmers having to cull animals or tourism operators losing business) but also the sense of alarm that biosecurity response operations evoke.^79^ The social and psychological impact of a biosecurity incursion on affected stakeholders is often underappreciated by government agencies coordinating a response.^80^^,^^81^ Including an understanding of the importance of citizen engagement in biosecurity responses may achieve a higher reduction in operational costs by factoring in voluntary actions of citizens or actions to protect livelihoods. It can also help build supportive social values and cooperation for future biosecurity responses. Given the similarity of such indirect effects, irrespective of whether the response is to a threat to human, animal, plant, or ecosystem health, a more integrated approach to assessing human wellbeing consequences to biosecurity responses should be considered.

The truly holistic approach in One Biosecurity requires thinking about how we can maintain a focus on wellbeing throughout the biosecurity continuum. For example, developing tools for the remediation of adverse effects of biosecurity actions can help ameliorate public resistance to control methods. A greater appreciation of how biosecurity intersects with wellbeing will help improve policies that plan to mitigate for adverse effects of interventions. It may also be key to curbing the dissemination of false and misleading information regarding biosecurity threats and interventions.

### Integrating surveillance and social data through sophisticated modeling

The multitude of quantitative models developed during the SARS-CoV-2 pandemic,^82^^,^^83^ has highlighted their utility for informing real-time decision-making in biosecurity response and planning. Unified decision support tools for human, animal, plant, and ecosystem health would deliver greater impacts than current sector-specific approaches, by ensuring that policy and management decisions are objective and optimized for the biosecurity system as a whole.^84^ For instance, a requirement across human, animal, plant, and ecosystem health is the need to provide proof-of-absence in a region from a specific invasive alien pathogen, pest, or weed, in order to facilitate trade by declaring area freedom or to support pest management by indicating eradication success.^85^ Proof-of-absence often relies on data from molecular or genomic eNA data and/or autonomous surveillance systems. Quantitative proof-of-absence decision support tools provide a robust framework for declaring the absence of a biosecurity threat given it was not detected during surveillance, and guide planning and optimization of risk-based surveillance strategies.^86^^,^^87^ Although systems of surveillance differ across human, animal, plant and ecosystem health sectors, the same underlying mathematical principles for estimating probability of absence apply, and there is great potential for wider implementation of the framework across sectors.^88^ Model developments to incorporate higher levels of biological and operational complexity, novel or integrated surveillance systems, and to facilitate application over larger spatial scales, will promote wider implementation across sectors. A unified proof-of-absence modeling approach for human, animal, plant, and ecosystem health will help ensure that policy and management decisions on when to declare absence of a biosecurity threat are objective, evidence-based, and cost-effective.

Advances in high-performance computing and statistical inference techniques are also improving the utility of agent-based models and related complex systems-based methods for modeling dynamics of holistic socio-environmental systems.^89^ Agent-based models (ABMs) are stochastic representations of a system, where multiple interacting agents and processes in an environment are simulated, and the evolution in numbers, state and/or location of agents is tracked over time.^90^ Multi-level ABMs, that represent multiple system levels (i.e., micro, meso, macro), offer rich insight into complex systems by revealing how local, individual-level interactions give rise to self-organization and emergent phenomena at a macroscale. While classical ABMs have been used for many years, they are frequently applied within sectors at smaller biological and spatial scales, such as host-pathogen dynamics.^91^^,^^92^ Complex systems-based modeling approaches, such as ABMs, could be better utilized in future in a whole socio-environmental system modeling approach, where agents and processes in human, animal, plant, and ecosystem health systems are represented, and at broad scale. Such models have been used to explore policy scenarios in realistic settings, for example to examine the role of invasive alien species management in the context of land-use dynamics and livelihood decisions.^93^

The accuracy of predictions from complex models and decision support tools depends on the availability and suitability of data to inform their many parameters. Artificial intelligence (AI) algorithms show promise to rapidly scan the large and complex data spread across multiple information sources (peer reviewed literature, government reports, blogs, and social media) and extract key information. To date, AI has been used with large datasets to predict the effect of climate scenarios on vector-borne animal diseases^94^ and to understand the epidemiology of the emergence of SARS-CoV-2.^95^^,^^96^ Thus, AI could be used across human, animal, plant and ecosystem health sectors to extract information to support complex modeling and horizon scanning. For example, artificial intelligence algorithms could gather, integrate, analyze, and visualize vast amounts of data from multiple unrelated sources supporting autonomous surveillance systems.^97^^,^^98^ However, key challenges that hinder data integration include how to effectively combine and analyze heterogeneous data collected by different sources and measured at different biological, spatial, and temporal scales; how to ensure data standardization and quality; how to maximize the opportunities of open data and the need for transparency, whilst balancing concerns for data security, sovereignty, and ethics. Overcoming these challenges to leverage data from multiple sources under a One Biosecurity approach will provide a more holistic view of human, animal, plant, and ecosystem health and maximize the collective impact of data.

## Future perspectives

**Figure 2:**
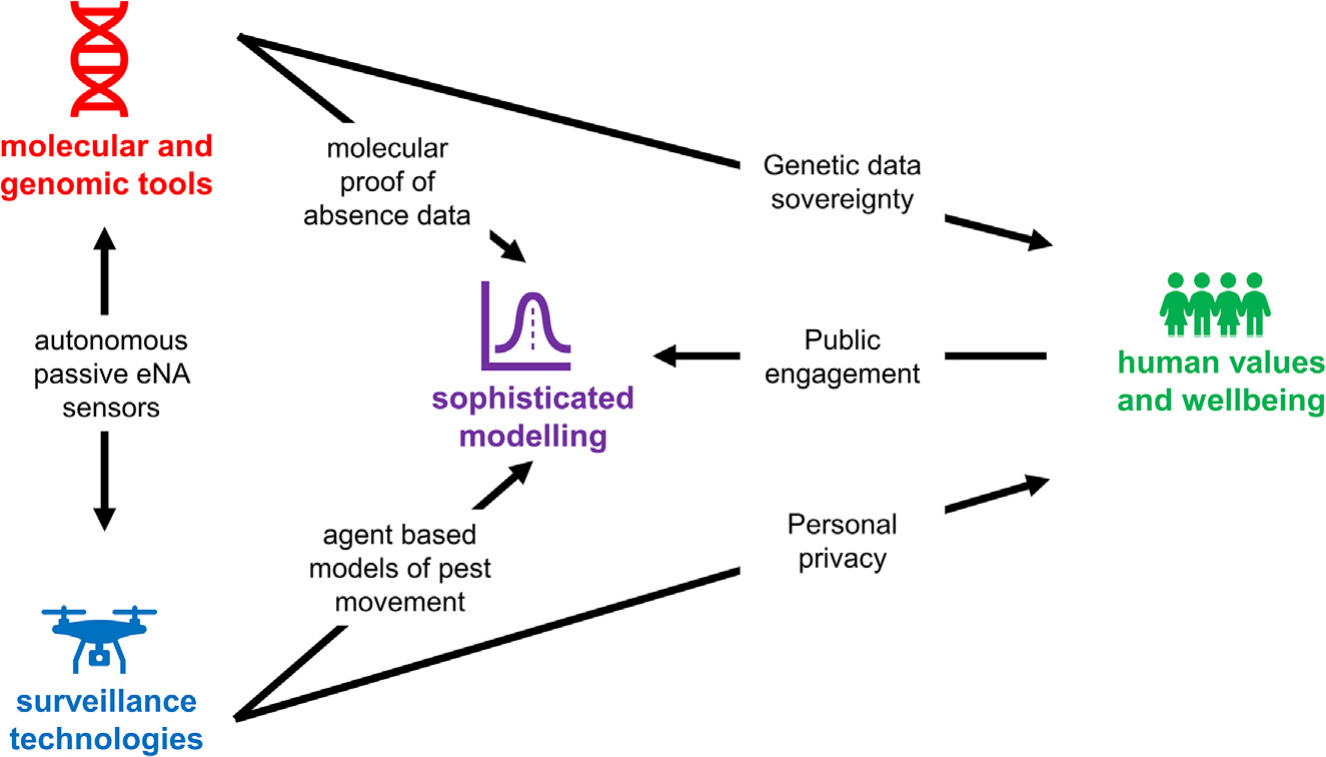
Schematic illustrating some of the interlinkages among the four emerging advances in biosecurity that underpin human, animal, plant, and ecosystem health Brief summaries of a selection of innovative approaches that link these four biosecurity areas (surveillance technologies, molecular and genomic tools, sophisticated modeling, human values and wellbeing) are provided for each link.

The preceding section highlights four emerging issues where an integrated One Biosecurity approach that spans human, animal, plant, and ecosystem health will bring considerable benefits for global and national biosecurity systems (Figure 2). Although the SARS-CoV-2 pandemic has been widely interpreted as a model for biosecurity responses to invasive alien pests, pathogens, and weeds,^99^^,^^100^^,^^101^ a detailed examination of what this might mean in practice has not yet been developed. By scanning the horizon for emerging issues that underpin human, animal, plant, and ecosystem health, it became clear that the SARS-CoV-2 pandemic has led to enhanced transdisciplinary research that is policy-relevant and has spawned the rapid implementation of new surveillance technologies, molecular and genomic tracing tools, modeling approaches as well as raising awareness of the importance of human values and wellbeing when responding to biosecurity threats. This momentum should not be lost, and it is essential that biosecurity systems begin to address these emerging issues. Furthermore, the applicability of these emerging issues and advancements across the spectrum of human, animal, plant, and ecosystem health highlights the importance of medics, veterinarians, epidemiologists, molecular biologists, plant pathologists, entomologists, ecologists, modelers, data scientists, social scientists, and holders of indigenous knowledge to jointly address these emerging issues.

One of the few silver linings of the SARS-CoV-2 pandemic was the global, transdisciplinary uniting of science and medicine to focus on a single global problem, sharing ideas, expertise, technology and information, from genomic studies, to tracking and tracing surveillance tools, spatiotemporal epidemiological models to forecast transmission trends, to the development of methods to limit SARS-CoV-2 spread and ultimately mitigate adverse impacts.^102^ Many researchers dropped the traditional siloed, competitive models to work together; fostered by publishers agreeing to make peer-reviewed research open access and implementing emergency measures to allow faster access to relevant information, resulting in almost 20,000 articles about SARS-CoV-2 shared in the first four months of the pandemic.^103^ The global community of researchers working on human, animal, plant, and ecosystem health do not yet have a similar collaborative ethos and remain strongly siloed in their single disciplines. Yet, emerging from the massive global effort to address the SARS-CoV-2 pandemic are insights into how to support and maintain productive global collaborative research including having clear rules for collaboration, open communication channels, prospects for joint funding, opportunities for face-to-face meetings at international workshops and symposia, as well as having a robust media presence focused on the specific research agenda.^104^

Furthermore, responding to future biosecurity threats before they arise will require a change to the governance of multilateral institutions to have a stronger focus on providing equal ownership and leadership opportunities to all member countries.^105^ A more strongly integrated and inclusive international research community together with a change to the global governance through the establishment of a specific biosecurity convention, is central to the concept of One Biosecurity.^11^

The SARS-CoV-2 pandemic also fueled digital transformation in health systems and demonstrated benefits of a more open data landscape.^106^ These advances should in the future facilitate the leveraging of environmental and ecological monitoring data, human health and socioeconomic data, and disease and pest surveillance data, to address complex biosecurity problems. Improving the efficiency of data access will help ensure that timely, reliable information is available for decision making, and for early detection/response to new biosecurity threats. Although the collation of surveillance data plays a crucial role in human, animal, plant, and ecosystem health, its use must be balanced carefully with ethical, legal, and social considerations. The SARS-CoV-2 pandemic resulted in an unprecedented scale of digital surveillance, particularly through contact tracing platforms that captured the location and mobility of individuals as well as in some cases facial recognition data.^107^ This level of data capture will likely pale into insignificance compared to the scale of information that might be collected in the future using autonomous sensors deployed using drones or robots.

In addition, the SARS-CoV-2 pandemic produced an extensive genetic testing infrastructure, developed in most countries to collect genomic data directly from individuals (e.g., nasal swabs) or indirectly in wastewater. There are concerns that this expensive technological infrastructure and supporting organizational backbones will be repurposed to conduct other forms of genetic testing to support wider goals of biosecurity.^108^ The use of surveillance technologies by government or business raises a number of pressing ethical concerns.^109^ Under such circumstances, tensions may exist between values associated with protecting human, animal, plant, and ecosystem health and those that relate to individual privacy, autonomy, and democratic accountability. Such data may be critical to the early detection of a biosecurity incursion, perhaps by mapping the likely spread of an invasive alien pathogen, pest, or weed. However, where such data enable tracing of movements of members of the public and associated metadata (including genomic information) that might be associated with biosecurity risk, then operating procedures must comply with country-specific laws or legislature that take into account ethical consideration and privacy (including genetic data) protection regimes.^110^ These ethical issues become all the more complex when biosecurity related data need to be shared internationally and research institutions require a commitment to open access to scientific information.^111^

These complexities highlight the importance of transparency, accountability, and community engagement (data governance) to ensure data usage is appropriate. Additionally, it is essential to establish the right to collect and use data (data sovereignty) through a process that has widespread community acceptance (social license). Consideration of the collation and use of data must include its potential to significantly impact on people’s lives (in particular the potential for a substantial impact on indigenous communities), requiring data systems to be co-designed and co-managed, and assuring that indigenous people will contribute to decision-making when preparing for and responding to an incursion.^112^

Biosecurity is intrinsically an outcome focused activity, where biosecurity systems are frequently judged on their failures (e.g., pest incursions, economic impacts) more than their successes (e.g., prevention of incursions, successful eradication). As a result, the value proposition for governments to invest in biosecurity is increasingly challenged by other more visible problems such as rising food and water insecurity,^113^ the need to reduce carbon emissions,^114^ improving the sustainability of agriculture,^115^ mitigating climate change impacts,^116^ and building resilience to natural disasters. Yet, the link between investing in biosecurity and reducing a nation’s exposure to such challenges are too infrequently understood by governments. For example, while the threat arising from emerging infectious diseases is now high on the policy agenda following the SARS-CoV-2 pandemic, the true value of the entire biosecurity system remains poorly quantified. However, in cases where a biosecurity system has been valued as a whole, it has revealed savings to national economies of the order of billions of dollars.^117^ Furthermore, a recent cost comparison found that economic losses from biological invasions were of similar magnitude to those arising from natural hazards, including storms, floods, and wildfires.^118^

Unfortunately, while national biosecurity systems were expected to be strengthened and modernized following the hard lessons from the SARS-CoV-2 pandemic, few countries have moved toward more technologically advanced surveillance, better data integration or fostered more resilient communities.^119^ As governments face challenges on all fronts, biosecurity systems that are streamlined across sectors are required to develop an enduring value proposition. Raising the profile of biosecurity both at a national and global level requires an integrated One Biosecurity approach that addresses the threats to human, animal, plant, and ecosystem health sectors. The four emerging areas describing new surveillance technologies, molecular and genomic tracing tools, modeling approaches, and raising awareness of the importance of human values and wellbeing can be extended across sectors to significantly improve the performance of the biosecurity system (Table 1). A One Biosecurity approach to detection, reporting and coordination of interventions, nationally and internationally, presents a stronger value proposition to governments,^11^ and will be more resilient against shifting priorities and limited resources.

**Table 1:** Illustrative examples of how each of the four underpinning biosecurity issues can be applied across the biosecurity continuum to manage offshore risk, boost border inspection, enhance incursion response, and improve long-term management of pathogens, pests, and weeds affecting the human, animal, plant, and ecosystem health sectors

Underpinning issue	Offshore risk reduction	Border inspection	Incursion response	Long-term management
Molecular and genomic tools	Phylodynamic analyses identify source regions of new pathovars	Environmental DNA to test compliance with ballast water regulations	Environmental DNA used to test waterways for invasive alien species	Genomic analysis to detect emergence of pesticide resistance
Human values and wellbeing	Trusted relationships created with trade partners	Raising international tourist compliance with biosecurity regulations	Improved engagement of citizen scientists for early detection of an incursion	Incorporating values of indigenous peoples in long-term management
Sophisticated modeling	Network models of global shipping traffic help profile biofouling risk	Stochastic models of species establishment risk based on interceptions	Agent-based models of invasive alien spread post-border	Proof of absence models used to declare eradication success
Surveillance technologies	Real-time tracking shipping containers and their contents	eNose used to detect pest odors in shipping containers	Tracking national-scale livestock movements using IoT	Realtime AI photo identification of invasive alien species in traps
