# Supplementary material for: Emerging advances in biosecurity to underpin human, animal, plant, and ecosystem health
Source: iScience. 2023 Jul 31;26(9):107462. doi: 10.1016/j.isci.2023.107462 (PMC10450416; doi:10.1016/j.isci.2023.107462)
Supplement: Document S1. Table S1 [file mmc1.pdf]

iScience, Volume *26*

Supplemental information

Emerging advances in biosecurity to underpin human, animal, plant, and ecosystem health

Philip E. Hulme, Jacqueline R. Beggs, Rachelle N. Binny, Jonathan P. Bray, Naomi Cogger, Manpreet K. Dhami, Susanna C. Finlay-Smits, Nigel P. French, Andrea Grant, Chad L. Hewitt, Eirian E. Jones, Phil J. Lester, and Peter J. Lockhart

Supplementary Materials

Table S1. Description of the synopsis titles considered in the horizon scan. Brief titles of the 34 synopses produced by participants listed in alphabetic order.

1. An integrated Biosecurity toolbox
2. Artificial intelligence for complicated data
3. Autonomous surveillance for biosecurity
4. Biosecurity risk assessment under changing climate
5. Comprehensive cost-benefit analysis
6. Cultural monitoring and surveillance
7. Do cryptic invasives pose an unrecognised risk?
8. Dogs as a screening test
9. Environmental nucleic acid (eNA) applications
10. Hidden pathogenic passengers. Microbiota
11. Human health and ecosystem health
12. Inclusivity in decision-making
13. Integrated data systems
14. Integrating knowledge of global change drivers
15. Invasive species and climate refugees
16. Molecular tools for mosquito borne pathogens
17. Multi-level agent-based modelling
18. Pesticide exposure
19. Predicting pathogen/ vector under climate change
20. Prediction of inherent invasiveness
21. Prioritisation and ranking invasion threats.
22. Proof of absence modelling framework
23. Real-time eDNA sequence analyses
24. Relational values and biosecure behaviour
25. Remediating adverse effects of biosecurity
26. Situational awareness of vectors
27. Social Media
28. Socio-ecological evolutionary colonisation
29. Te Ao Māori
30. Tracing/tracking using Internet of Things

31. Understanding pathogen origins using phylodynamics
32. Vector borne pathogens.
33. Wellbeing and biosecurity restrictions
34. What are “successful” biosecurity systems?
